# Binding of *Kingella kingae* RtxA Toxin Depends on Cell Surface Oligosaccharides, but Not on β_2_ Integrins

**DOI:** 10.3390/ijms21239092

**Published:** 2020-11-29

**Authors:** Waheed Ur Rahman, Adriana Osickova, Nela Klimova, Jinery Lora, Nataliya Balashova, Radim Osicka

**Affiliations:** 1 Institute of Microbiology of the Czech Academy of Sciences, Videnska 1083, 142 20 Prague 4, Czech Republic; waheed.rahman@biomed.cas.cz (W.U.R.); osickova@biomed.cas.cz (A.O.); nelaklim@biomed.cas.cz (N.K.); 2 Department of Biochemistry, Faculty of Science, Charles University in Prague, Hlavova 8, 128 43 Prague 2, Czech Republic; 3 Department of Basic and Translational Sciences, School of Dental Medicine, University of Pennsylvania, 240 S. 40th St., Philadelphia, PA 19104, USA; jinery.lora@pennmedicine.upenn.edu (J.L.); natbal@upenn.edu (N.B.)

**Keywords:** β_2_ integrins, *Kingella kingae*, oligosaccharides, RtxA, RTX toxin

## Abstract

The Gram-negative coccobacillus *Kingella kingae* is increasingly recognized as an important invasive pediatric pathogen that causes mostly bacteremia and skeletal system infections. *K. kingae* secretes an RtxA toxin that belongs to a broad family of the RTX (Repeats in ToXin) cytotoxins produced by bacterial pathogens. Recently, we demonstrated that membrane cholesterol facilitates interaction of RtxA with target cells, but other cell surface structures potentially involved in toxin binding to cells remain unknown. We show that deglycosylation of cell surface structures by glycosidase treatment, or inhibition of protein N- and O-glycosylation by chemical inhibitors substantially reduces RtxA binding to target cells. Consequently, the deglycosylated cells were more resistant to cytotoxic activity of RtxA. Moreover, experiments on cells expressing or lacking cell surface integrins of the β_2_ family revealed that, unlike some other cytotoxins of the RTX family, *K. kingae* RtxA does not bind target cells via the β_2_ integrins. Our results, hence, show that RtxA binds cell surface oligosaccharides present on all mammalian cells but not the leukocyte-restricted β_2_ integrins. This explains the previously observed interaction of the toxin with a broad range of cell types of various mammalian species and reveals that RtxA belongs to the group of broadly cytolytic RTX hemolysins.

## 1. Introduction

The Gram-negative coccobacillus *Kingella kingae* is a member of the commensal oropharyngeal flora of young children and, until recently, it was believed to be a rare pathogen [1,2,3]. However, improvements in culture techniques and molecular detection methods have resulted in recognition of *K. kingae* as a leading cause of osteomyelitis and septic arthritis in children [1,3,4,5,6,7]. Other invasive diseases caused by *K. kingae* include bacteremia, endocarditis, meningitis, pneumonia, ocular infections, peritonitis, or pericarditis [7,8].

*K. kingae* secretes the RtxA toxin that is cytotoxic to synovial cells, bone osteosarcoma cells, macrophage-like cells, and respiratory epithelial cells [9,10], suggesting that the toxin might play an important role in the pathogenic process. Indeed, experiments with an RtxA-deficient mutant KKNB100 in an infant rat model revealed that RtxA is a critical virulence factor of *K. kingae* [11]. RtxA belongs to the RTX (Repeats in ToXin) family of pore-forming cytotoxins that are produced by many Gram-negative bacterial pathogens, including the genera *Actinobacillus*, *Aggregatibacter, Bordetella*, *Escherichia, Mannheimia*, *Moraxella*, *Morganella*, *Pasteurella*, *Proteus*, and *Vibrio* [12]. Sequence homology with the RTX toxins revealed four functional segments in the 956 residues-long RtxA: (i) a pore-forming domain encompassing residues ~140 to 410, harboring four putative transmembrane α-helices; (ii) an acylated segment, where the RtxA protoxin (proRtxA) is posttranslationally activated; recently, we experimentally demonstrated that the acyltransferase RtxC activates proRtxA by fatty acyl modification on lysine residues 558 and 689, primarily with myristoyl and hydroxymyristoyl chains [9,13]; (iii) a typical calcium-binding RTX domain between residues ~730 to 810, harboring the conserved repetitions of a nonapeptide motif X-(L/I/F)-X-G-G-X-G-(N/D)-D (where X is any amino acid residue), which form calcium-binding sites and (iv) a C-terminal secretion signal. Upon binding to target cells that is facilitated by membrane cholesterol, RtxA inserts itself into the cell membrane and forms cation-selective membrane pores, which induce cation flux leading to cell death [9,14].

Based on cellular specificity, the pore-forming RTX cytotoxins can be roughly divided into two different groups: (i) hemolysins, capable of lysing erythrocytes and exhibiting toxicity towards a wide range of cell types from various species; and (ii) leukotoxins that exhibit narrow cell type and species specificity due to cell-specific binding through the β_2_ integrins expressed on the cell surface of leukocytes [12]. The β_2_ integrins include four heterodimeric transmembrane glycoproteins composed of a common β_2_ subunit, CD18, and one of the variable α subunits, α_L_ (CD11a), α_M_ (CD11b), α_X_ (CD11c), or α_D_ (CD11d) [15]. The *A. actinomycetemcomitans* leukotoxin (LtxA) is specific for human leukocytes and interacts with the CD11a/CD18 integrin [16]. The *M. haemolytica* leukotoxin (LktA) specifically targets bovine leukocytes, and it was initially shown to bind most of the β_2_ integrins, very likely via their common CD18 subunit [17,18]. However, later findings revealed that only CD11a/CD18 is involved in LktA-induced biological effects [19,20]. The *Escherichia coli* α-hemolysin (HlyA) was also found to bind leukocytes through CD11a/CD18 [16], but a later report indicated that the CD11a/CD18 integrin is not a receptor for HlyA [21]. Finally, the CyaA toxin from *B. pertussis* has been shown to use the integrin CD11b/CD18 as a specific receptor on myeloid phagocytes [22,23,24].

Nevertheless, CyaA and HlyA also appear to be somewhat promiscuous and exhibit a detectable cytotoxic activity on a wide spectrum of cells from various species that lack the β_2_ integrins on the cell surface, such as erythrocytes, endothelial or epithelial cells from mice, ruminants, and primates, respectively [12]. Similarly, LtxA also exhibits a detectable hemolytic activity on human and sheep erythrocytes [25]. Our results with the CyaA, HlyA, and LtxA toxins showed that they exhibit a weak lectin activity and interact with the oligosaccharide chains of their β_2_ integrin receptors [26,27]. This raised the possibility that the binding of the RTX leukotoxins to the cells lacking the β_2_ integrins and binding of the RTX hemolysins to various cells might primarily occur through the recognition of glycosylated membrane components, such as glycoproteins or gangliosides.

Here, we demonstrate that binding of RtxA to the cell surface depends on oligosaccharides, but not on the leukocyte-restricted β_2_ integrins. Thus, our results explain previously observed broad cellular specificity of RtxA [9,10] and show that the toxin belongs to the group of broadly cytolytic RTX hemolysins.

## 2. Results

### 2.1. Pretreatment of Cells with Glycosidases Reduces Binding of RtxA

**Figure 1:**
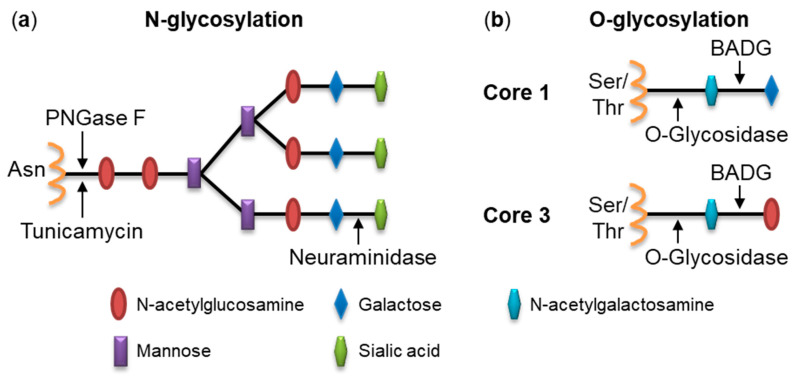
Simplified schematic representation of N- and O-linked oligosaccharide chains. (**a**) a representative complex type of oligosaccharide chain linked to an asparagine residue of an N-glycosylation site (Asn-Xaa-Ser/Thr). Cleavage sites for highly specific glycosidases PNGase F and neuraminidase are depicted by black arrows. Chemical inhibitor tunicamycin blocks N-glycosylation of newly-synthesized proteins at asparagine residues (black arrow); (**b**) schematic representation of Core 1 and Core 3 O-linked (Ser/Thr) disaccharides that are specifically removed from glycoproteins by O-glycosidase (black arrows). BADG acts as a competitive inhibitor of O-glycan chain extension.

To analyze the potential importance of cell surface glycosylation for purified RtxA binding and cytotoxicity, we used several types of selective glycosidases able to remove oligosaccharide chains from cell surface structures: (i) a neuraminidase that hydrolyzes α-(2→3), α-(2→6), α-(2→8)-glycosidic linkages of terminal sialic residues in glycoproteins and glycolipids; (ii) a peptide-N-glycosidase F (PNGase F) that cleaves the amide bond between the N-acetylglucosamine and asparagine residue of N-linked glycoproteins; and (iii) an endo-α-N-acetylgalactosaminidase (O-glycosidase) that catalyzes the removal of Core 1 and Core 3 O-linked disaccharides from glycoproteins (Figure 1). These enzymes were used to deglycosylate model target cells, whose susceptibility to RtxA was demonstrated previously, such as the laryngeal HLaC-78 squamous cells, hypopharyngeal FaDu epithelial cells, bone osteosarcoma U-2 OS epithelial cells, synovial sarcoma SW 982 cells and monocytic THP-1 cells, respectively [9]. After a 1-h preincubation of cells at 37 °C with neuraminidase, PNGase F, or O-glycosidase, the cells were washed, then incubated with the purified Dy495-labeled RtxA protein (2 µg/mL) at 4 °C for different times and binding of the toxin to the cell surface was determined by flow cytometry.

**Figure 2:**
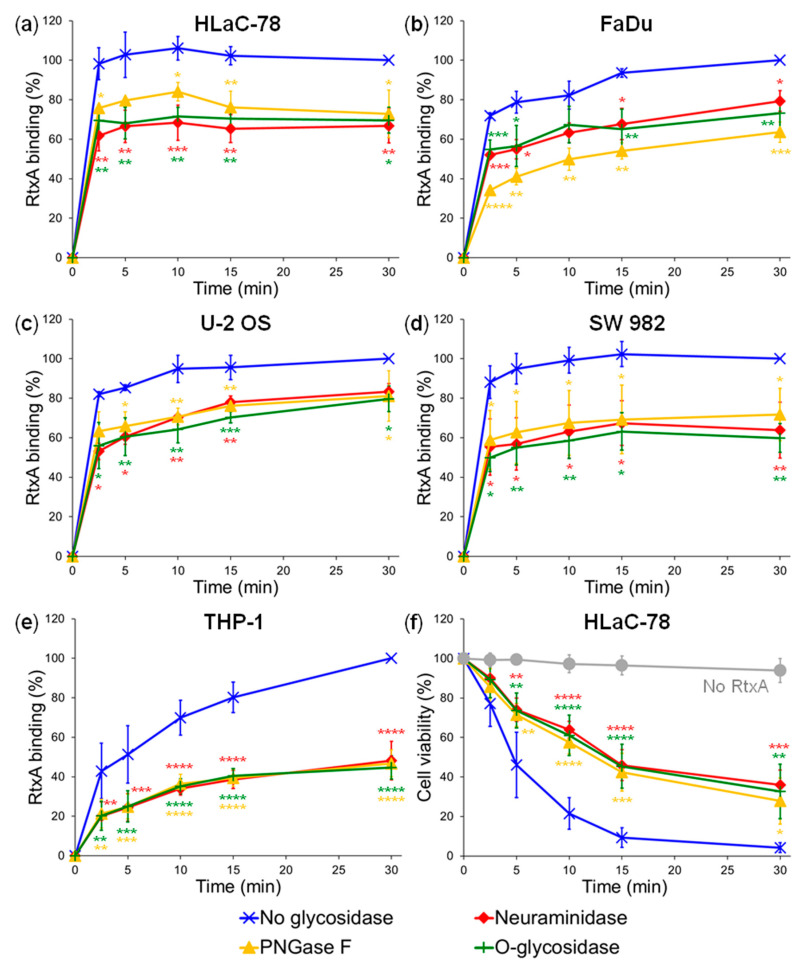
Deglycosylation of cells decreases binding and cytotoxic activity of RtxA. Human HLaC-78 (**a**), FaDu (**b**), U-2 OS (**c**), SW 982 (**d**), and THP-1 (**e**) cells were treated with neuraminidase, PNGase F, O-glycosidase, or buffer alone (no glycosidase) for 1 h at 37 °C. Then, the cells (1 × 10^6^/mL) were incubated with 2 µg/mL of the purified and labeled RtxA-Dy495 toxin for different times at 4 °C and the surface-bound RtxA-Dy495 was determined by flow cytometry. Binding data were deduced from the MFI values and expressed as percentage of RtxA binding to glycosidase-untreated cells at time 30 min (taken as 100%). Each point represents the mean value ± SD of at least three independent experiments. Significant differences between mean values of RtxA binding to glycosidase-untreated and treated cells are shown (*, *p* < 0.05; **, *p* < 0.01; ***, *p* < 0.001; ****, *p* < 0.0001; ANOVA). (**f**) Glycosidase-treated and untreated HLaC-78 cells (1 × 10^6^/mL) were incubated with 0.5 µg/mL of RtxA for different times at 37 °C. Cell viability was determined by a cell viability staining assay using 1 μg/mL of Hoechst 33258 followed by flow cytometry. The viability of cells incubated without RtxA (time 0) was reported as 100%. Each point represents the mean value ± SD of five independent experiments. Significant differences between mean values of cell viability of glycosidase-treated and untreated cells upon incubation with RtxA are indicated (*, *p* < 0.05; **, *p* < 0.01; ***, *p* < 0.001; ****, *p* < 0.0001; ANOVA).

As shown in Figure 2a–e, binding of RtxA was highest in glycosidase-untreated cells, while toxin binding was significantly reduced in all tested cell types when sialic residues of the cell surface structures were removed by neuraminidase. The highest ~50% decrease of RtxA binding (expressed as a percentage of RtxA binding to glycosidase-untreated cells) was observed in neuraminidase-treated THP-1 cells at all analyzed time points (Figure 2e), while the decrease of RtxA binding to other neuraminidase-treated cells varied between ~25 and 40% (Figure 2a–d). Moreover, when RtxA was preincubated with 10 mM sialic acid before addition to cells, the inhibition of RtxA binding was observed and reached 24% on HLaC-78 cells and 32% on THP-1 monocytes (Figure S1). The decrease of RtxA binding between ~22 and 43% was then observed when N-linked oligosaccharide chains of cell surface glycoproteins were removed by PNGase F or when O-linked oligosaccharides were removed by O-glycosidase (Figure 2a–e). Further substantial decrease of RtxA binding to HLaC-78 cells (66%) and THP-1 monocytes (77%) was observed, when the cells were deglycosylated by a combination of PNGase F and O-glycosidase (Figure S2). All these results demonstrate that binding of RtxA to different cell types was considerably reduced upon deglycosylation of cell surface structures, suggesting that binding of RtxA to cell surface depends on its glycosylation status.

To confirm the physiological relevance of cell surface glycans for cytotoxic action of RtxA on cells, the susceptibility of deglycosylated HLaC-78 cells to RtxA-mediated killing was determined by a cell viability assay using Hoechst 33258 nucleic acid stain followed by flow cytometry as previously described [9]. As shown in Figure 2f, glycosidase-untreated HLaC-78 cells were substantially more susceptible to the RtxA-mediated killing than HLaC-78 cells that were deglycosylated by neuraminidase, PNGase F, or O-glycosidase. While RtxA (0.5 µg/mL) reduced cell viability of glycosidase-untreated cells within 30 min of incubation at 37 °C to ~5%, about 40% of the glycosidase-treated HLaC-78 cells survived over that period (Figure 2f). These results show that cytotoxic activity of RtxA also depends on the glycosylation of target cells.

### 2.2. Inhibition of Protein Glycosylation Reduces Binding of RtxA to Cells

To corroborate that binding of RtxA to cells depends on glycosylated cell surface structures, HLaC-78, FaDu, U-2 OS, SW 982, and THP-1 cells were treated for 24 h prior to binding experiments with: (i) 10 µg/mL of tunicamycin, an antibiotic that blocks N-glycosylation of newly-synthesized proteins at asparagine residues, or with (ii) 2 mM benzyl-2-acetamido-2-deoxy-α-D-galactopyranoside (BADG), a competitive inhibitor of O-glycan chain extension (Figure 1). The inhibitor-treated cells were washed, incubated with purified Dy495-labeled RtxA (2 µg/mL) at 4 °C for different times and binding of the toxin to cells was determined by flow cytometry.

**Figure 3:**
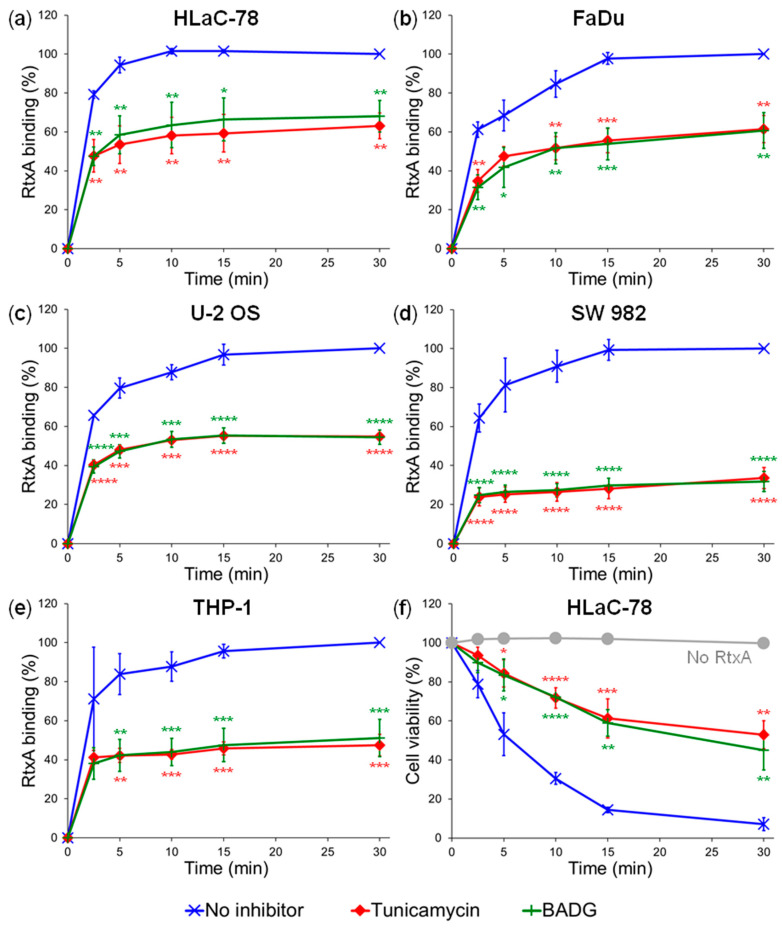
Treatment of cells with different inhibitors of glycosylation decreases binding and cytotoxic activity of RtxA. Human HLaC-78 (**a**), FaDu (**b**), U-2 OS (**c**), SW 982 (**d**), and THP-1 (**e**) cells were treated with tunicamycin, benzyl-2-acetamido-2-deoxy-α-D-galactopyranoside (BADG) or buffer alone (no inhibitor) for 24 h at 37 °C. Then, the cells (1 × 10^6^/mL) were incubated with 2 µg/mL of the purified and labeled RtxA-Dy495 toxin for different times at 4 °C, and the surface-bound RtxA-Dy495 was determined by flow cytometry. Binding data were deduced from the MFI values and expressed as percentage of RtxA binding to untreated cells at time 30 min (taken as 100%). Each point represents the mean value ± SD of three independent experiments. Significant differences between mean values of RtxA binding to inhibitor-untreated cells and cells treated with inhibitors are indicated (*, *p* < 0.05; **, *p* < 0.01; ***, *p* < 0.001; ****, *p* < 0.0001; ANOVA). (**f**) Inhibitor-treated and untreated HLaC-78 cells (1 × 10^6^/mL) were incubated with 0.5 µg/mL of the purified RtxA toxin for different times at 37 °C. Cell viability was determined by a cell viability staining assay using 1 μg/mL of Hoechst 33258 followed by flow cytometry. The viability of cells incubated without RtxA (time 0) was reported as 100%. Each point represents the mean value ± SD of three independent experiments. Significant differences between mean values of cell viability of inhibitor-treated and inhibitor-untreated cells upon incubation with RtxA are indicated (*, *p* < 0.05; **, *p* < 0.01; ***, *p* < 0.001; ****, *p* < 0.0001; ANOVA).

As summarized in Figure 3a–e, the inhibition of N-glycosylation of cell surface proteins resulted in ~40 to 70% decrease of RtxA binding to tunicamycin-treated cells, as compared to inhibitor-untreated cells. The inhibition of O-glycosylation of cell surface proteins reduced RtxA binding to BADG-treated cells by ~35 to 70% (Figure 3a–e). Hence, both enzymatic and inhibitor-mediated deglycosylation of cell surface structures caused a loss of RtxA binding with comparable efficacy.

Similarly to cells treated with glycosidases, the HLaC-78 cells pretreated with the inhibitors of N- and O- glycosylation were less sensitive to cytotoxic activity of RtxA. As demonstrated in Figure 3f, RtxA (0.5 µg/mL) reduced cell viability of inhibitor-treated HLaC-78 cells within 30 min of incubation at 37 °C to ~50%, while only about 5% of the untreated cells survived over that period (Figure 3f). These results provide further evidence that cytotoxic activity of RtxA depends on the glycosylation status of target cells.

### 2.3. β_2_ Integrins Are Not Cellular Receptors of RtxA

**Figure 4:**
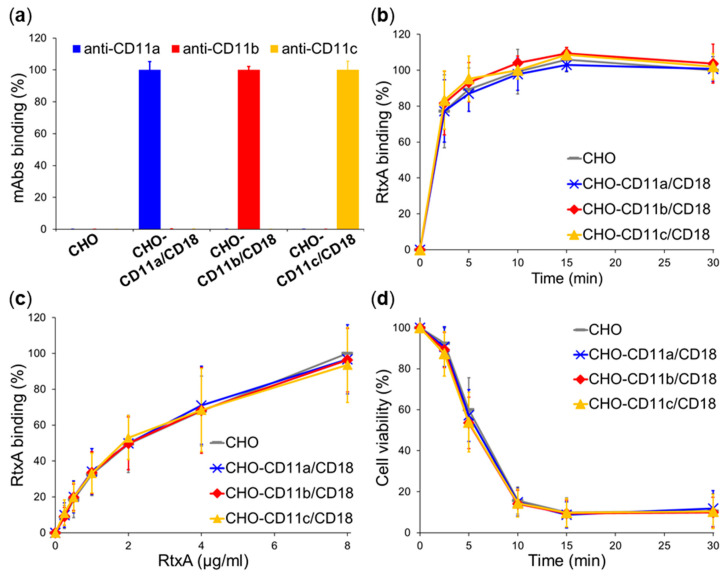
RtxA binds and kills cells expressing β_2_ integrins with the same efficacy as cells lacking any β_2_ integrin. (**a**) Stably transfected CHO cells expressing the human integrin CD11a/CD18, CD11b/CD18, or CD11c/CD18, or mock-transfected CHO cells expressing no β_2_ integrin (1 × 10^6^/mL) were incubated for 30 min at 4 °C with anti-CD11a, anti-CD11b, and anti-CD11c mAbs and analyzed by flow cytometry. Binding data were deduced from the MFI values and the highest MFI value for each mAb was taken as 100%. (**b**) CHO cells expressing β_2_ integrin molecules, or no β_2_ integrin (1 × 10^6^/mL) were incubated with 2 µg/mL of the purified and labeled RtxA-Dy495 toxin for different times at 4 °C and analyzed by flow cytometry. (**c**) CHO cells expressing β_2_ integrin molecules, or no β_2_ integrin (1 × 10^6^/mL) were incubated with different concentrations of RtxA-Dy495 for 30 min at 4 °C and analyzed by flow cytometry. (**b**,**c**) Binding data were deduced from the MFI values and expressed as percentage of RtxA binding to mock-transfected CHO cells at time 30 min (**b**) or at a concentration of 8 µg/mL (**c**) (taken as 100%). Each point represents the mean value ± SD of four independent experiments. RtxA binding to integrin-expressing cells was at all measured time and concentration points statistically indistinguishable from binding to mock-transfected CHO cells expressing no β_2_ integrin (*p*-value > 0.05; ANOVA). (**d**) CHO cells expressing β_2_ integrin molecules, or no β_2_ integrin (1 × 10^6^/mL) were incubated with 0.5 µg/mL of the purified RtxA toxin for different times at 37 °C. Cell viability was determined by a cell viability staining assay using 1 μg/mL of Hoechst 33258 followed by flow cytometry. The viability of cells incubated without RtxA (time 0) was taken as 100%. Each point represents the mean value ± SD of four independent experiments. Upon incubation with RtxA, the viability of β_2_ integrin-expressing CHO cells was at all measured time points found to be statistically the same as the viability of CHO cells expressing no β_2_ integrin (*p*-value > 0.05; ANOVA).

It has previously been demonstrated that the RTX toxins LtxA of *A. actinomycetemcomitans* and LktA of *M. haemolytica* specifically interact with target cells through the β_2_ integrin CD11a/CD18 [16,19,20] and that the RTX cytotoxin CyaA of *B. pertussis* efficiently recognizes the β_2_ integrin CD11b/CD18 on myeloid phagocytes [22,23]. To investigate whether RtxA specifically interacts with the β_2_ integrin(s), we examined binding and cytotoxicity of RtxA on Chinese hamster ovary (CHO) cells that were stably transfected to individually express three distinct human β_2_ integrins, CD11a/CD18, CD11b/CD18, or CD11c/CD18 (Figure 4a). These cells were previously constructed by our group to demonstrate that CyaA specifically binds the integrin CD11b/CD18 but does not bind the integrins CD11a/CD18 or CD11c/CD18 [23].

As shown in Figure 4b, the β_2_ integrin-expressing CHO transfectants, incubated with purified Dy495-labeled RtxA (2 µg/mL) at 4 °C, bound at each time point similar amounts of the toxin as the mock-transfected CHO cells expressing no β_2_ integrin. Similarly, when the cells were exposed at 4 °C for 30 min to different concentrations of the highly purified toxin (0–8 µg/mL), no differences in RtxA binding to integrin- and mock-transfected CHO cells were observed over a range of toxin concentrations (Figure 4c). Finally, the viability of the β_2_ integrin-expressing CHO transfectants was reduced in the same time-dependent manner upon treatment with RtxA (0.5 µg/mL) at 37 °C as the viability of mock-transfected CHO cells (Figure 4d). All of these results indicate that the β_2_ integrins are not specifically recognized by the RtxA toxin on the surface of CHO cells.

To corroborate this observation, we analyzed RtxA binding to primary mouse macrophages by flow cytometry. Bone marrow cells (BMCs) were isolated from CD11a knockout (KO), CD11b KO and control (WT) C57BL/6 mice and differentiated into mature macrophages in the presence of macrophage colony-stimulating factor (M-CSF). The presence of WT and KO *ITGAL* (encoding CD11a) and *ITGAM* (encoding CD11b) alleles was verified by genotyping (Figure S3).

**Figure 5:**
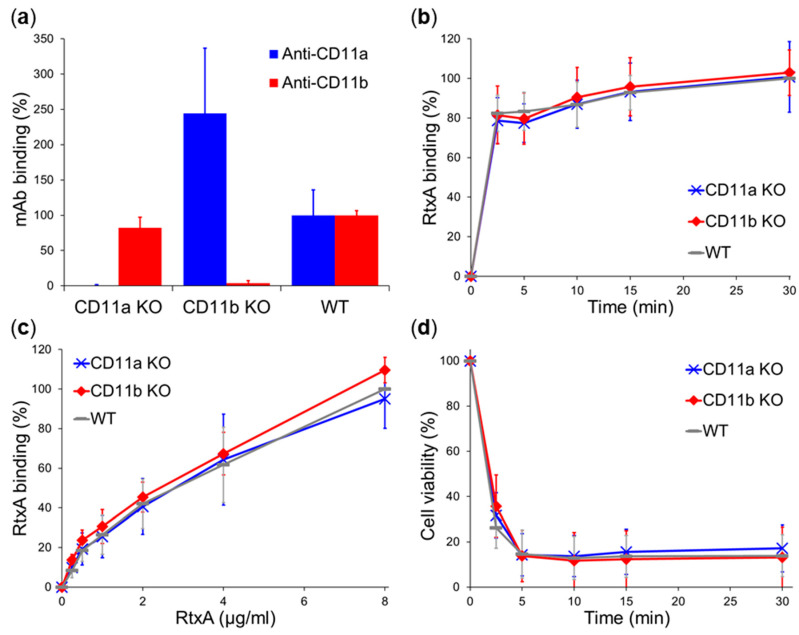
RtxA binds and kills CD11a KO and CD11b KO macrophages with the same efficacy as WT macrophages. (**a**) CD11a KO, CD11b KO, and control WT mouse macrophages (1 × 10^6^/mL) were incubated for 30 min at 4 °C with anti-CD11a and anti-CD11b mAbs and analyzed by flow cytometry. Binding data were deduced from the MFI values and expressed as percentage of mAb binding to WT macrophages (taken as 100%). (**b**) CD11a KO, CD11b KO, and WT macrophages (1 × 10^6^/mL) were incubated with 2 µg/mL of the purified and labeled RtxA-Dy495 toxin for different times at 4 °C and analyzed by flow cytometry. (**c**) CD11a KO, CD11b KO, and WT macrophages (1 × 10^6^/mL) were incubated with different concentrations of RtxA-Dy495 for 30 min at 4 °C and analyzed by flow cytometry. (**b**,**c**) Binding data were deduced from the MFI values and expressed as percentage of RtxA binding to WT macrophages at time 30 min (**b**) or at a concentration of 8 µg/mL (**c**) (taken as 100%). Each point represents the mean value ± SD of at least three independent experiments. RtxA binding to CD11a KO and CD11b KO macrophages was at all measured time and concentration points statistically indistinguishable from binding to WT macrophages (*p*-value > 0.05; ANOVA). (**d**) CD11a KO, CD11b KO, and WT macrophages (1 × 10^6^/mL) were incubated with 0.5 µg/mL of RtxA for different times at 37 °C. Cell viability was determined by a cell viability staining assay using 1 μg/mL of Hoechst 33258 followed by flow cytometry. The viability of cells incubated without RtxA (time 0) was taken as 100%. Each point represents the mean value ± SD of at least five independent experiments. Upon incubation with RtxA, the viability of CD11a KO and CD11b KO macrophages was at all measured time points found to be statistically the same as the viability of WT macrophages (*p*-value > 0.05; ANOVA).

As expected, an anti-CD11a monoclonal antibody (mAb) did not recognize CD11a KO macrophages and an anti-CD11b mAb did not bind CD11b KO cells, while both mAbs recognized WT macrophages (Figure 5a). As summarized in Figure 5b, CD11a KO and CD11b KO macrophages, incubated with purified Dy495-labeled RtxA (2 µg/mL) at 4 °C, bound at each time similar amounts of the toxin as WT macrophages expressing both CD11a and CD11b integrins. In addition, no significant differences in RtxA binding to CD11a KO, CD11b KO, and WT macrophages were observed upon incubation of the cells with different concentrations of the highly purified toxin (0–8 µg/mL) at 4 °C for 30 min (Figure 5c). In contrast, CyaA specifically recognizing CD11b/CD18 bound CD11b KO macrophages with ~4 times lower efficacy than WT macrophages (Figure S4). Finally, the viability of CD11a KO and CD11b KO macrophages was decreased in the same time-dependent manner upon incubation with RtxA (0.5 µg/mL) at 37 °C as the viability of WT macrophages (Figure 5d). These results confirm that, in contrast to some other RTX toxins, the RtxA cytotoxin of *K. kingae* does not recognize the β_2_ integrins CD11a/CD18 and CD11b/CD18 as specific receptors on the surface of macrophages.

## 3. Discussion

We have previously demonstrated that the RTX cytotoxin CyaA of *B. pertussis* interacts with N-linked oligosaccharide chains of the β_2_ integrin CD11b/CD18 located in the C-terminal part of the CD11b subunit [26,27]. CyaA binding to CD11b/CD18-expressing J774A.1 macrophage-like cells, human neutrophils, and CHO-CD11b/CD18 cells was reduced by ~70 to 80%, when the cells were pretreated with neuraminidase that removes peripheral sialic acid residues from glycoproteins [26]. A near complete inhibition (~90 to 95%) of CyaA binding to the CD11b/CD18-expressing cells was then observed when oligosaccharide chains were completely removed by PNGase F, or when N-glycosylation of *de novo* synthesized proteins was inhibited by tunicamycin [26]. We have further revealed that this initial interaction of CyaA with N-linked glycan chains of CD11b is followed by high-affinity toxin binding to a proteinaceous segment containing residues 614 to 682 of CD11b [23]. We have proposed that the N-linked oligosaccharide chains and the segment 614–682 of CD11b may together form a highly organized structure that accounts for the affinity and specificity of CyaA-integrin interaction and enables subsequent biological activities of the toxin [27].

Here, we show that binding and cytotoxic activities of *K. kingae* RtxA toxin depend on glycosylated surface structures of various cells, but not on the leukocyte-restricted and highly-glycosylated β_2_ integrins, as previously shown for CyaA and some other RTX toxins [16,19,20,22,23]. Binding and cytotoxicity of RtxA could be significantly reduced when the cells were preincubated with neuraminidase (Figure 2), removing peripheral sialic acid residues from glycosylated molecules. This suggests that the sialic acid residues covalently linked to various cell surface structures (glycoproteins, gangliosides) are important for binding and cytotoxic activities of RtxA, similarly to the requirement for terminal sialic acid residues of N-linked oligosaccharide chains of the integrin CD11b/CD18 enabling activities of CyaA [26]. We have previously proposed that the interaction between CD11b/CD18 and CyaA would be mediated by the peripheral negatively charged sialic acid residues of the integrin and the positively charged arginine and lysine residues of the RTX domain of CyaA [26], where the main integrin–interaction segment of CyaA is located between residues 1166–1281 [28]. Despite the fact that RtxA also harbors an RTX domain [9,10] that might be involved in the interaction of the toxin with the sialic acid residues of the cell surface structures, further experiments are needed to confirm this hypothesis.

To investigate the contribution of N- or O-linked oligosaccharide chains of the cell surface glycoproteins to the binding and cytotoxic activities of RtxA, we used here both enzymatic (PNGase F, O-glycosidase) and inhibitor-mediated (tunicamycin, BADG) deglycosylation of various cells. Deglycosylated cells bound significantly lower amounts of RtxA and were subsequently more resistant to the cytotoxic effect of the toxin in comparison with untreated cells (Figure 2 and Figure 3). These results indicate that RtxA recognizes, aside from the sialic acid residues, other saccharide units of the oligosaccharide complexes of the cell surface glycoproteins.

Recently, we employed several different methods to show that cholesterol is important for binding and cytotoxicity of RtxA [9]. Hence, cholesterol, a key structural component of all animal cell membranes, and the oligosaccharide complexes at the cell surfaces jointly act as the main structures used by the RtxA toxin for the interaction with cell membranes.

Previously, we observed that human THP-1 monocytes expressing β_2_ integrins were more resistant to the cytotoxic action of RtxA than human cells lacking β_2_ integrins (HLaC-78, and FaDu) [9], which suggested that RtxA might not recognize the β_2_ integrin(s). Using CHO cells stably transfected with human β_2_ integrins, we confirmed here that RtxA bound and killed the β_2_ integrin-expressing CHO transfectants with the same efficacy as mock-transfected CHO cells that did not express β_2_ integrins (Figure 4). For comparison, when the same CHO transfectants were previously used to study the CyaA interaction with the β_2_ integrins, we demonstrated that CHO-CD11b/CD18 cells bound about two orders of magnitude more CyaA toxin than the CHO-CD11a/CD18 and CHO-CD11c/CD18 cells or the mock-transfected cells expressing no β_2_ integrin at all [23].

To further confirm that RtxA binds to cells independently on the CD11a/CD18 and CD11b/CD18 integrins that are specifically recognized by some other RTX toxins [16,19,20,22,23], we used macrophages derived from BMCs isolated from CD11a KO and CD11b KO mice. Indeed, RtxA bound to CD11a KO and CD11b KO macrophages with the same efficacy as to WT macrophages (Figure 5). For comparison, CyaA bound CD11b KO macrophages with ~4 times lower efficacy than WT macrophages (Figure S4). In addition, RtxA binding to CHO cells (Figure 4c) and macrophages (Figure 5c) was not saturable up to a high toxin concentration of 8 µg/mL. This further indicates that RtxA binds cells in a rather unspecific manner and exhibits promiscuous binding via various cell surface glycoproteins and glycolipids.

In conclusion, we demonstrate that binding and cytotoxic activities of the RtxA toxin produced by *K. kingae* depend on recognition of glycans on target cell surface structures, but not on cell surface β_2_ integrins. Since glycoproteins and glycolipids are present on the surface of all mammalian cells, these results explain the previously observed promiscuity of RtxA interaction with various cell types from different mammalian species [9,10]. This reveals that the RtxA toxin belongs to the group of broadly cytolytic RTX hemolysins.

## 4. Materials and Methods

### 4.1. Antibodies

FITC-labeled mAbs specific for human CD11a (MEM-25) and CD11c (BU15), for mouse CD11a (M17/4) and CD11b (M1/70) and FITC-labeled rat IgG2b (RTG2B1-2) isotype control were purchased from Exbio (Vestec, Czech Republic). OKM1 mAb recognizing human CD11b was isolated from the OKM1 hybridoma purchased from the European Collection of Cell Cultures, Porton Down, UK. PerCP-eF710-labeled mAb specific for mouse CD11a (M17/4) and PerCP-eF710- and FITC-labeled rat IgG2a (eBR2a) isotype controls were obtained from eBioscience (San Diego, CA, USA).

### 4.2. Cell Lines and Growth Conditions

Human laryngeal HLaC-78 cells were established from a laryngeal squamous cell carcinoma by Zenner et al. [29]. Human hypopharyngeal FaDu cells (ATCC HTB-43), human bone osteosarcoma U-2 OS epithelial cells (ATCC HTB-96), human synovial sarcoma SW 982 cells (ATCC HTB-93), and human monocytic THP-1 cells (ATCC TIB-202) were purchased from the American Type Culture Collection (ATCC, Manassas, VA, USA). HLaC-78, FaDu and THP-1 cells were grown in RPMI 1640 (Sigma-Aldrich, St. Louis, MO, USA) with 10% fetal calf serum (FCS) (GIBCO Invitrogen, Grand Island, NY, USA) and antibiotic antimycotic solution (1000 U/mL penicillin, 0.1 mg/mL streptomycin and 0.25 mg/mL amphotericin; Sigma-Aldrich, St. Louis, MO, USA). U-2 OS and SW 982 cells were cultured in Dulbecco’s Modified Eagle’s medium (DMEM, Sigma-Aldrich, St. Louis, MO, USA) with 10% FCS and antibiotic antimycotic solution.

CHO-K1 Chinese hamster ovary cells (ATCC CCL-61) stably transfected with human CD11a/CD18, CD11b/CD18, CD11c/CD18, or mock transfected were prepared previously [23] and grown in F12K medium (GIBCO Invitrogen, Grand Island, NY, USA) supplemented with 10% FCS and antibiotic antimycotic solution.

### 4.3. Animal Studies

The CD11a KO mouse strain (LFA-1 KO; JAX stock #005257) [30], the CD11b KO mouse strain (B6.129S4-Itgamtm1Myd/J; JAX stock #003991) [31], and the C57BL/6J mouse control strain (WT; JAX stock #000664) were purchased from the Jackson Laboratory, Sacramento, CA, USA. The animals were housed and bred in pathogen-free conditions at the University of Pennsylvania. All animal experiments were approved by Animal Welfare Committee at the University of Pennsylvania (the Animal Welfare Assurance Number A3079-01) and performed under Animal protocol number 806760 approved on August 27, 2019.

### 4.4. Isolation of Bone Marrow Cells and Their Differentiation to Macrophages

BMCs were obtained from the tibias and femurs of 8–10-week-old mice. The whole body of euthanized animals was disinfected by soaking in 70% ethanol for 10 min. The limbs were dissected and stored on ice in PBS containing 1000 U/mL penicillin-streptomycin (Thermo Fisher Scientific, Waltham, MA, USA). Then, the muscle and connective tissue from tibia and femur were removed, bones were washed, and the edges of the tibia and femur were cut below the ending of the marrow cavity. The 27G 1/2 inch needle attached to a 10 mL syringe was inserted to the bone opening and BMCs were flushed out from the marrow cavity using BMC growth medium (α-MEM medium containing 10% FCS and 1000 U/mL penicillin-streptomycin). The cell suspension was filtered through a 70 mm filter mesh strainer to remove any bone spicules, muscle, and cell clumps and centrifuged at 1000× *g* for 10 min. The resulting cell pellet was resuspended in 1xRBC Lysis Buffer (GIBCO Invitrogen, Grand Island, NY, USA) and BMCs cells were collected by centrifugation. The BMCs cell pellet was resuspended in BMC growth medium and incubated in a T-25 culture dish for 6–12 h at 37 °C under 5% CO_2_. Non-adherent BMCs cells were collected, resuspended in RPMI 1640 supplemented with 10% FCS, antibiotic antimycotic solution and 10% L929 cell-conditioned, M-CSF-containing supernatant and differentiated to macrophages for 7–10 days at 37 °C under 5% CO_2_.

The presence of WT and KO *ITGAL* (encoding CD11a) and *ITGAM* (encoding CD11b) alleles was verified by genotyping according to protocols provided by Jackson Laboratory, Sacramento, CA, USA.

### 4.5. Protein Production, Purification, and Labeling

The recombinant RtxC-activated RtxA toxin was expressed from the pT7*rtxC*-*rtxA* plasmid in the *E. coli* strain BL21/pMM100 (Novagen, Madison, WI, USA) and purified under denaturing conditions in urea by a combination of affinity chromatography on an Ni-NTA agarose column (Qiagen, Germantown, MD, USA) and hydrophobic chromatography on a Phenyl-Sepharose CL-4B column (Sigma–Aldrich, St. Louis, MO, USA) as previously described [9]. On-column labeling of RtxA with Dy495-NHS ester (Dyomics, Jena, Germany) was performed on the Phenyl-Sepharose column after the Ni-NTA agarose purification step [9].

The CyaC-acylated CyaA toxoid (CyaA-AC^-^, unable to convert ATP to cAMP) was expressed from the pT7CACT1-derived plasmid [32] using the *E. coli* strain XL1-Blue (Stratagene, La Jolla, CA, USA). The protein was purified by a combination of ion exchange chromatography on DEAE-Sepharose CL-6B (Sigma–Aldrich, St. Louis, MO, USA) and hydrophobic chromatography on Phenyl-Sepharose CL-4B (Sigma–Aldrich, St. Louis, MO, USA) as previously described [32]. On-column labeling of the toxoid with Dy647-NHS ester (Dyomics, Jena, Germany) was performed on the Phenyl-Sepharose column after the DEAE-Sepharose purification step [23].

### 4.6. Deglycosylation of Cell Surface Structures with Glycosidases

Cultured cells were collected, washed in HEPES-buffered salt solution (HBSS buffer; 10 mM HEPES (pH 7.4), 140 mM NaCl, 5 mM KCl) complemented with 2 mM CaCl_2_, 2 mM MgCl_2_ (HBSS-Ca/Mg buffer), and diluted with the same buffer to 1 × 10^6^ cells/mL. The cells were incubated for 1 h at 37 °C with 50 mU/mL neuraminidase, 500 mU/mL of peptide-N-glycosidase F (PNGase F), or 40 mU/mL of endo-α-N-acetylgalactosaminidase (O-glycosidase; all three enzymes were purchased from New England Biolabs, Ipswich, MA, USA), or in buffer alone (no glycosidase control). After treatment, the cells were washed with HBSS-Ca/Mg buffer and used in experiments analyzing cell binding and cytotoxicity of RtxA.

### 4.7. Inhibition of Glycosylation Using Chemical Inhibitors

Cultured cells (1 × 10^6^) were treated with 10 µg/mL of tunicamycin (Sigma-Aldrich, St. Louis, MO, USA) or 2 mM benzyl-2-acetamido-2-deoxy-α-D-galactopyranoside (BADG; Sigma-Aldrich, St. Louis, MO, USA) at 37 °C for 24 h in growth medium with 10% FCS. Then, the cells were harvested, washed with HBSS-Ca/Mg buffer and used to analyze cell binding and cytotoxicity of RtxA.

### 4.8. RtxA Binding to Cells

1 × 10^6^ cells/mL in HBSS-Ca/Mg buffer were incubated with 2 µg/mL of labeled RtxA-Dy495 (directly 100-fold diluted into a suspension of cells from the toxin stock in 50 mM Tris-HCl (pH 8.0) and 8 M urea) for different times (0–30 min) at 4 °C. The cell-bound RtxA-Dy495 was determined by flow cytometry using a FACS LSR II instrument (BD Biosciences, San Jose, CA, USA) in the presence of 1 µg/mL of the nucleic acid stain Hoechst 33258. Data were processed with the FlowJo software (Tree Star, Ashland, OR, USA) and appropriate gatings were used to exclude debris, cell aggregates, and dead cells (Hoechst 33258-positive staining). Binding data were deduced from the mean fluorescence intensity (MFI) of RtxA-Dy495 and expressed as a percentage of the toxin binding to untreated cells in time 30 min: (MFI value of glycosidase- or inhibitor-treated or untreated cells incubated with RtxA-Dy495 − MFI value of glycosidase- or inhibitor-treated or untreated cells incubated without RtxA-Dy495 (time 0))/(MFI value of untreated cells incubated with RtxA-Dy495 (time 30 min) − MFI value of untreated cells incubated without RtxA-Dy495 (time 0)) × 100.

### 4.9. Cytotoxicity Assay

Cell viability in the presence of RtxA was determined as previously described [9]. Briefly, 1 × 10^6^ cells/mL in HBSS-Ca/Mg buffer were incubated with 0.5 µg/mL of purified RtxA (directly 100-fold diluted into a suspension of cells from the toxin stock in 50 mM Tris-HCl (pH 8.0) and 8 M urea) for different times at 37 °C. Cell viability was determined by a vital dye staining using 1 µg/mL of the nucleic acid stain Hoechst 33258 followed by flow cytometry using a FACS LSR II instrument (BD Biosciences, San Jose, CA, USA). Data were analyzed with the FlowJo software (Tree Star, Ashland, OR, USA) and appropriate gatings were done to exclude cell aggregates, debris, dying cells and dead cells (Hoechst 33258-positive staining). Cell viability was calculated by using the formula: (count of viable cells in a sample with RtxA/count of viable cells in a sample without RtxA) × 100.

### 4.10. Statistical Analysis

Results were presented as the arithmetic mean ± standard deviation (SD) of the mean. Statistical significance was calculated by Student’s *t*-test when two groups were compared or by one-way ANOVA followed by Dunnett’s post-test when more than two groups were compared (GraphPad Prism 8.0; GraphPad Software, La Jolla, CA, USA).

## Supplementary Materials

Supplementary Materials can be found at https://www.mdpi.com/1422-0067/21/23/9092/s1. Figure S1: Binding of RtxA to cells is partially inhibited by free sialic acid, Figure S2: RtxA binding to cells deglycosylated with a combination of PNGase F and O-glycosidase was substantially decreased compared to cells deglycosylated individually with PNGase F or O-glycosidase, Figure S3: Genotyping of bone marrow-derived macrophages, Figure S4: Bone marrow-derived macrophages of CD11b KO mice bind substantially lower amounts of CyaA than macrophages of WT mice.

## Abbreviations

BADG	Benzyl-2-acetamido-2-deoxy-α-D-galactopyranoside
BMC	Bone marrow cell
CHO	Chinese hamster ovary
mAb	Monoclonal antibody
M-CSF	Macrophage colony-stimulating factor
MFI	Mean fluorescence intensity
O-glycosidase	Endo-α-N-acetylgalactosaminidase
PNGase	Peptide-N-glycosidase F
RTX	Repeats in toxin

## References

[1] Yagupsky P. (2015). *Kingella kingae*: Carriage, transmission, and disease. Clin. Microbiol. Rev..

[2] Ceroni D., Dubois-Ferriere V., Cherkaoui A., Lamah L., Renzi G., Lascombes P., Wilson B., Schrenzel J. (2013). 30 years of study of *Kingella kingae*: Post tenebras, lux. Future Microbiol..

[3] Principi N., Esposito S. (2015). *Kingella kingae* infections in children. BMC Infect. Dis..

[4] Gene A., Garcia-Garcia J.J., Sala P., Sierra M., Huguet R. (2004). Enhanced culture detection of *Kingella kingae*, a pathogen of increasing clinical importance in pediatrics. Pediatr. Infect. Dis. J..

[5] Moumile K., Merckx J., Glorion C., Berche P., Ferroni A. (2003). Osteoarticular infections caused by *Kingella kingae* in children: Contribution of polymerase chain reaction to the microbiologic diagnosis. Pediatr. Infect. Dis. J..

[6] Verdier I., Gayet-Ageron A., Ploton C., Taylor P., Benito Y., Freydiere A.M., Chotel F., Berard J., Vanhems P., Vandenesch F. (2005). Contribution of a broad range polymerase chain reaction to the diagnosis of osteoarticular infections caused by *Kingella kingae*: Description of twenty-four recent pediatric diagnoses. Pediatr. Infect. Dis. J..

[7] Dubnov-Raz G., Ephros M., Garty B.Z., Schlesinger Y., Maayan-Metzger A., Hasson J., Kassis I., Schwartz-Harari O., Yagupsky P. (2010). Invasive pediatric *Kingella kingae* Infections: A nationwide collaborative study. Pediatr. Infect. Dis. J..

[8] Yagupsky P., Porsch E., St Geme J.W. (2011). Kingella kingae: An emerging pathogen in young children. Pediatrics.

[9] Osickova A., Balashova N., Masin J., Sulc M., Roderova J., Wald T., Brown A.C., Koufos E., Chang E.H., Giannakakis A. (2018). Cytotoxic activity of *Kingella kingae* RtxA toxin depends on post-translational acylation of lysine residues and cholesterol binding. Emerg. Microbes Infect..

[10] Kehl-Fie T.E., St Geme J.W. (2007). Identification and characterization of an RTX toxin in the emerging pathogen *Kingella kingae*. J. Bacteriol..

[11] Chang D.W., Nudell Y.A., Lau J., Zakharian E., Balashova N.V. (2014). RTX toxin plays a key role in *Kingella kingae* virulence in an infant rat model. Infect. Immun..

[12] Linhartova I., Bumba L., Masin J., Basler M., Osicka R., Kamanova J., Prochazkova K., Adkins I., Hejnova-Holubova J., Sadilkova L. (2010). RTX proteins: A highly diverse family secreted by a common mechanism. FEMS Microbiol. Rev..

[13] Osickova A., Khaliq H., Masin J., Jurnecka D., Sukova A., Fiser R., Holubova J., Stanek O., Sebo P., Osicka R. (2020). Acyltransferase-mediated selection of the length of the fatty acyl chain and of the acylation site governs activation of bacterial RTX toxins. J. Biol. Chem..

[14] Barcena-Uribarri I., Benz R., Winterhalter M., Zakharian E., Balashova N. (2015). Pore forming activity of the potent RTX-toxin produced by pediatric pathogen *Kingella kingae*: Characterization and comparison to other RTX-family members. Biochim. Biophys. Acta.

[15] Mazzone A., Ricevuti G. (1995). Leukocyte CD11/CD18 integrins: Biological and clinical relevance. Haematologica.

[16] Lally E.T., Kieba I.R., Sato A., Green C.L., Rosenbloom J., Korostoff J., Wang J.F., Shenker B.J., Ortlepp S., Robinson M.K. (1997). RTX toxins recognize a beta2 integrin on the surface of human target cells. J. Biol. Chem..

[17] Ambagala T.C., Ambagala A.P., Srikumaran S. (1999). The leukotoxin of *Pasteurella haemolytica* binds to beta(2) integrins on bovine leukocytes. FEMS Microbiol. Lett..

[18] Li J., Clinkenbeard K.D., Ritchey J.W. (1999). Bovine CD18 identified as a species specific receptor for *Pasteurella haemolytica* leukotoxin. Vet. Microbiol..

[19] Jeyaseelan S., Hsuan S.L., Kannan M.S., Walcheck B., Wang J.F., Kehrli M.E., Lally E.T., Sieck G.C., Maheswaran S.K. (2000). Lymphocyte function-associated antigen 1 is a receptor for *Pasteurella haemolytica* leukotoxin in bovine leukocytes. Infect. Immun..

[20] Thumbikat P., Dileepan T., Kannan M.S., Maheswaran S.K. (2005). Characterization of *Mannheimia* (*Pasteurella*) *haemolytica* leukotoxin interaction with bovine alveolar macrophage beta2 integrins. Vet. Res..

[21] Valeva A., Walev I., Kemmer H., Weis S., Siegel I., Boukhallouk F., Wassenaar T.M., Chavakis T., Bhakdi S. (2005). Binding of *Escherichia coli* hemolysin and activation of the target cells is not receptor-dependent. J. Biol. Chem..

[22] Guermonprez P., Khelef N., Blouin E., Rieu P., Ricciardi-Castagnoli P., Guiso N., Ladant D., Leclerc C. (2001). The adenylate cyclase toxin of *Bordetella pertussis* binds to target cells via the alpha(M)beta(2) integrin (CD11b/CD18). J. Exp. Med..

[23] Osicka R., Osickova A., Hasan S., Bumba L., Cerny J., Sebo P. (2015). *Bordetella* adenylate cyclase toxin is a unique ligand of the integrin complement receptor 3. Elife.

[24] Wald T., Osickova A., Masin J., Liskova P.M., Petry-Podgorska I., Matousek T., Sebo P., Osicka R. (2016). Transmembrane segments of complement receptor 3 do not participate in cytotoxic activities but determine receptor structure required for action of *Bordetella* adenylate cyclase toxin. Pathog. Dis..

[25] Balashova N.V., Crosby J.A., Al Ghofaily L., Kachlany S.C. (2006). Leukotoxin confers beta-hemolytic activity to *Actinobacillus actinomycetemcomitans*. Infect. Immun..

[26] Morova J., Osicka R., Masin J., Sebo P. (2008). RTX cytotoxins recognize beta2 integrin receptors through N-linked oligosaccharides. Proc. Natl. Acad. Sci. USA.

[27] Hasan S., Osickova A., Bumba L., Novak P., Sebo P., Osicka R. (2015). Interaction of *Bordetella* adenylate cyclase toxin with complement receptor 3 involves multivalent glycan binding. FEBS Lett..

[28] El-Azami-El-Idrissi M., Bauche C., Loucka J., Osicka R., Sebo P., Ladant D., Leclerc C. (2003). Interaction of *Bordetella pertussis* adenylate cyclase with CD11b/CD18: Role of toxin acylation and identification of the main integrin interaction domain. J. Biol. Chem..

[29] Zenner H.P., Lehner W., Herrmann I.F. (1979). Establishment of carcinoma cell lines from larynx and submandibular gland. Arch. Otorhinolaryngol..

[30] Ding Z.M., Babensee J.E., Simon S.I., Lu H., Perrard J.L., Bullard D.C., Dai X.Y., Bromley S.K., Dustin M.L., Entman M.L. (1999). Relative contribution of LFA-1 and Mac-1 to neutrophil adhesion and migration. J. Immunol..

[31] Coxon A., Rieu P., Barkalow F.J., Askari S., Sharpe A.H., von Andrian U.H., Arnaout M.A., Mayadas T.N. (1996). A novel role for the beta 2 integrin CD11b/CD18 in neutrophil apoptosis: A homeostatic mechanism in inflammation. Immunity.

[32] Osicka R., Osickova A., Basar T., Guermonprez P., Rojas M., Leclerc C., Sebo P. (2000). Delivery of CD8(+) T-cell epitopes into major histocompatibility complex class I antigen presentation pathway by *Bordetella pertussis* adenylate cyclase: delineation of cell invasive structures and permissive insertion sites. Infect Immun..

