# Supplementary material for: Binding of Kingella kingae RtxA Toxin Depends on Cell Surface Oligosaccharides, but Not on β2 Integrins
Source: Int J Mol Sci. 2020 Nov 29;21(23):9092. doi: 10.3390/ijms21239092 (PMC7730106; doi:10.3390/ijms21239092)
Supplement: Supplementary file 1 [file ijms-21-09092-s001.pdf]

Supporting Information: Binding of *Kingella kingae* RtxA Toxin Depends on Cell Surface Oligosaccharides, but Not on β2 Integrins

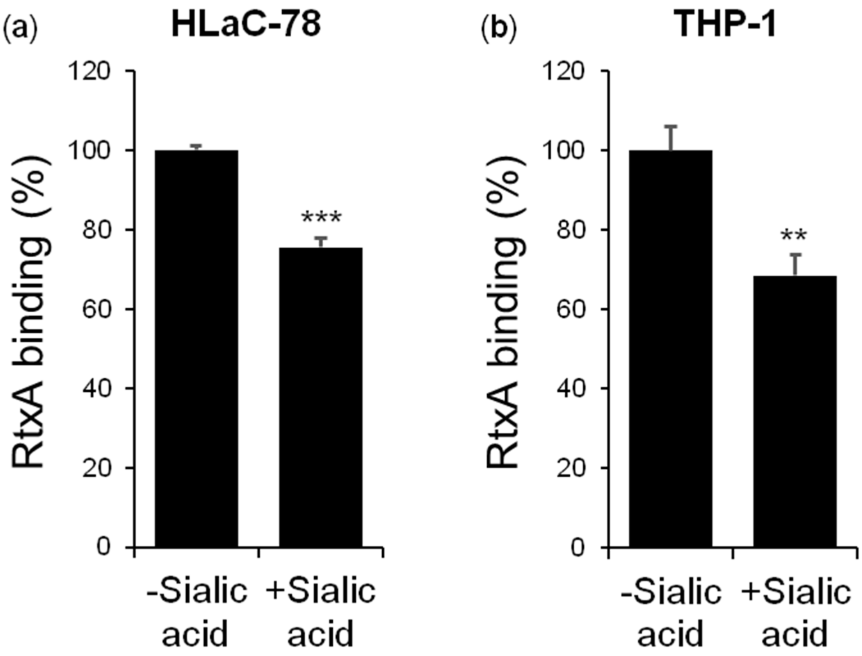

Figure S1. Binding of RtxA to cells is partially inhibited by free sialic acid. The purified and labeled RtxA-Dy495 toxin (2 μg/ml) was preincubated with 10 mM sialic acid or HBSS-Ca/Mg buffer alone for 1 min and then the solutions were added to HLaC-78 (**a**) or THP-1 (**b**) cells (1×10^6/ml). After 10 min at 4°C, the surface-bound RtxA-Dy495 was determined by flow cytometry and binding data were deduced from the MFI values and expressed as percentage of RtxA binding to sialic acid-untreated cells (taken as 100%). Each bar represents the mean value with SD of three independent experiments (**, $p<0.01$; ***, $p<0.001$; Student's t-test).

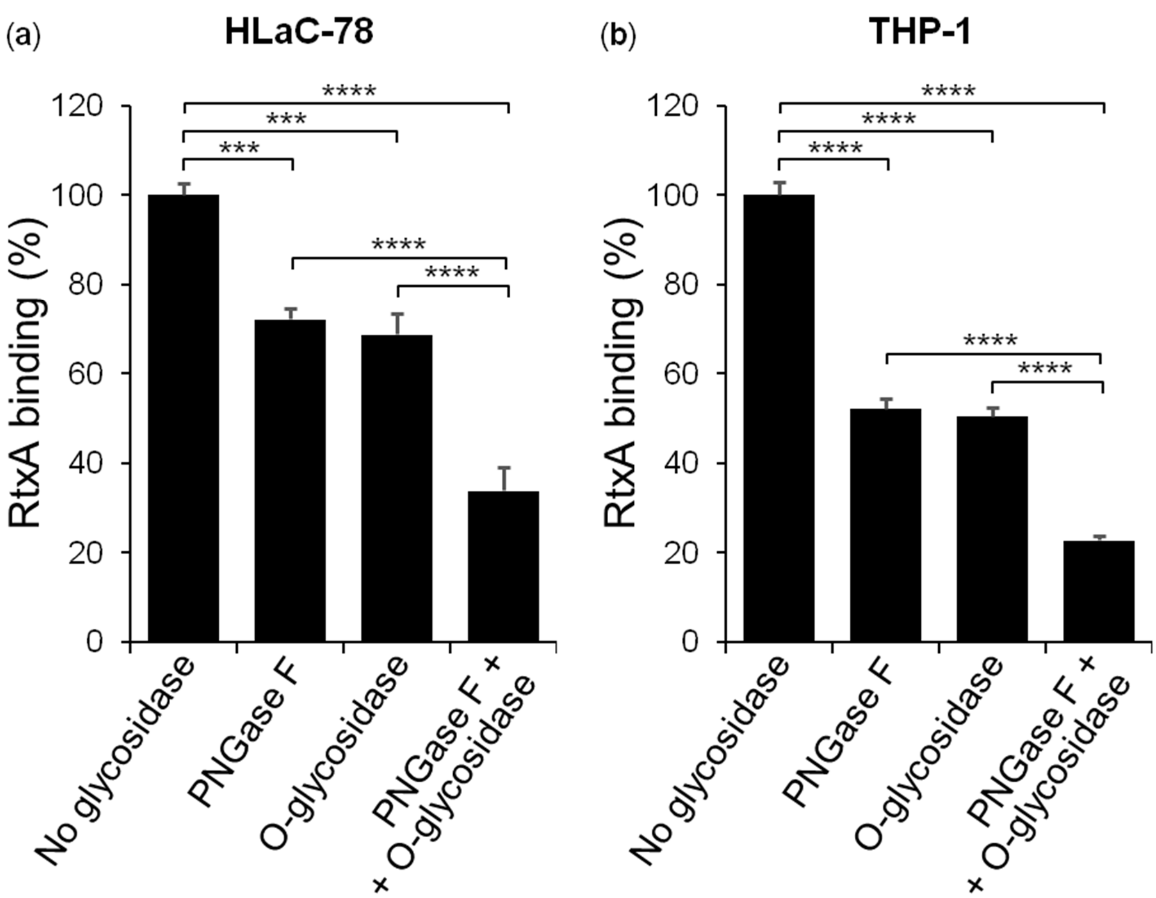

Figure S2. RtxA binding to cells deglycosylated with a combination of PNGase F and O-glycosidase was substantially decreased compared to cells deglycosylated individually with PNGase F or O-glycosidase. Human HLaC-78 (**a**) and THP-1 (**b**) cells were treated with PNGase F, O-glycosidase, a combination of PNGase F and O-glycosidase or buffer alone (no glycosidase) for 1 h at 37 °C. Then the cells (1×10^6/ml) were incubated with 2 μg/ml of the purified and labeled RtxA-Dy495 toxin for 10 min at 4 °C and the surface-bound RtxA-Dy495 was determined by flow cytometry. Binding data were deduced from the MFI values and expressed as percentage of RtxA binding to glycosidase-untreated cells (taken as 100%). Each bar represents the mean value with SD of three independent experiments (***, p<0.001; ****, p<0.0001; ANOVA).

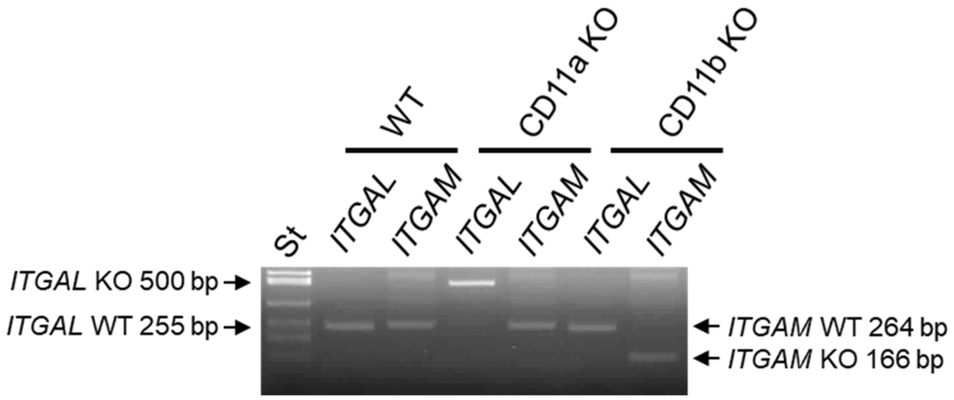

Figure S3. Genotyping of bone marrow-derived macrophages. DNA isolated from 1×10^6 bone marrow-derived macrophages of WT, CD11a KO and CD11b KO mice was amplified by PCR with primer pairs specific for *ITGAL* (encoding CD11a) and *ITGAM* (encoding CD11b) alleles. The product size of *ITGAL* and *ITGAM* WT alleles is 255 bp and 264 bp, respectively, of *ITGAL* KO allele is 500 bp, and of *ITGAM* KO allele is 166 bp.

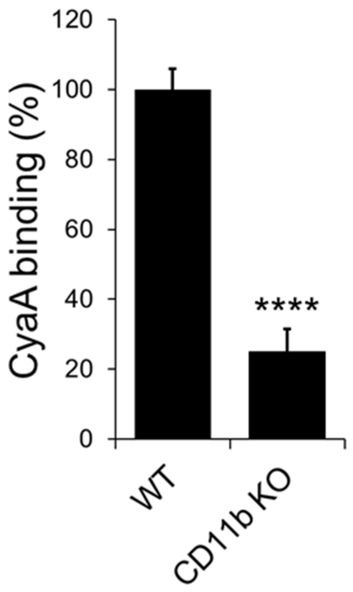

Figure S4. Bone marrow-derived macrophages of CD11b KO mice bind substantially lower amounts of CyaA than macrophages of WT mice. CD11b KO and WT macrophages (1×10^6/ml) were incubated with 5 μg/ml of the purified and Dy647-labeled CyaA toxoid (CyaA-AC$^-$, unable to covert ATP to cAMP) for 30 min at 4 °C and analyzed by flow cytometry. Binding data were deduced from the MFI values and expressed as percentage of CyaA binding to WT macrophages (taken as 100%). Each bar represents the mean value with SD of at least five independent experiments (****, p-value < 0.0001; Student's t-test).
